# Comparative Assessment of Two Commercial Real-Time PCR Assays for the Diagnosis of *Trypanosoma cruzi* DNA in Serum

**DOI:** 10.3390/microorganisms11040901

**Published:** 2023-03-30

**Authors:** Simone Kann, Gustavo Concha, Felix Weinreich, Andreas Hahn, Christian Rückert, Jörn Kalinowski, Olfert Landt, Hagen Frickmann

**Affiliations:** 1 Medical Mission Institute, 97074 Würzburg, Germany; 2 Organization Wiwa Yugumaiun Bunkauanarrua Tayrona (OWYBT), Department Health Advocacy, Valledupar 2000001, Colombia; 3 Department of Microbiology and Hospital Hygiene, Bundeswehr Hospital Hamburg, 20359 Hamburg, Germany; 4 Institute for Medical Microbiology, Virology and Hygiene, University Medicine Rostock, 18057 Rostock, Germany; 5 CeBiTec Centrum for Biotechnology, Bielefeld University, 33615 Bielefeld, Germany; 6 TIB Molbiol, 12103 Berlin, Germany

**Keywords:** Chagas, diagnostic, molecular detection, test comparison, Colombia, sensitivity, specificity

## Abstract

This study was performed to comparably assess two commercial real-time PCR assays for the identification of *Trypanosoma cruzi* DNA in serum. A total of 518 Colombian serum samples with high pre-test probability for infections with either *T. cruzi* or apathogenic *Trypanosoma rangeli* were assessed. The assessment comprised the NDO real-time PCR (TIB MOLBIOL, ref. no. 53-0755-96, referred to as the TibMolBiol assay in the following) with specificity for *T. cruzi* and the RealStar Chagas PCR Kit 1.0 (altona DIAGNOSTICS, order no. 611013, referred to as the RealStar assay in the following) targeting a kinetoplast sequence of both *T. cruzi* and *T. rangeli* without further discrimination. To discriminate between *T. cruzi*- and *T. rangeli*-specific real-time PCR amplicons, Sanger sequencing results were available for a minority of cases with discordant real-time PCR results, while the amplicons of the remaining discordant samples were subjected to nanopore sequencing. The study assessment indicated a proportion of 18.1% (n = 94) *T. cruzi*-positive samples next to 24 samples (4.6%) containing DNA of the phylogenetically related but apathogenic parasite *T. rangeli*. The observed diagnostic accuracy as expressed by sensitivity and specificity was 97.9% (92/94) and 99.3% (421/424) with the TibMolBiol assay and 96.8% (91/94) and 95.0% (403/424) with the RealStar assay, respectively. Reduced specificity resulted from cross-reaction with *T. rangeli* in all instances (3 cross-reactions with the TibMolBiol assay and 21 cross-reactions with the RealStar assay). DNA from the six discrete typing units (DTUs) of *T. cruzi* was successfully amplified by both real-time PCR assays. In summary, both assays showed a comparable diagnostic accuracy for the diagnosis of *T. cruzi* from human serum, with a slightly higher specificity seen for the TibMolBiol assay. The pronounced co-amplification of DNA from apathogenic *T. rangeli* according to the RealStar assay may be a disadvantage in areas of co-circulation with *T. cruzi*, while the test performance of the two compared assays will be quite similar in geographic settings where *T. rangeli* infections are unlikely.

## 1. Introduction

Although Chagas disease caused by *Trypanosoma cruzi* is well known as a poverty-related tropical infection [1] and common case definitions include serology based on two different assays as the diagnostic approach of choice [2,3], increasing availability of modern real-time PCR technology even in resource-limited settings has facilitated an interest in molecular diagnostic approaches targeting *T. cruzi* in the recent years. So, traditional diagnostic assays and those based on real-time PCR were introduced and comparably assessed with both human and environmental samples [4,5,6,7,8,9,10,11,12]. While most of the introduced assays were in-house designed, the commercial satellite DNA-targeting RealCycler CHAG kit (EMELCA Bioscience, Clinge, The Netherlands) showed a perfect specificity of 100% with a still-improvable sensitivity of 7.14% in a recent assessment [12]. The kit’s sensitivity was further dependent on optimized nucleic acid extraction [10]. Of note, the sensitivity of satellite DNA-based real-time PCR could be increased by adding an additional kinetoplast DNA-based assay [12]; however, this would be at the cost of lower specificity due to pronounced cross-reactivity of kinetoplast DNA-based assays with the DNA of apathogenic *Trypanosoma rangeli* [9]. In contrast to the effect of the choice of the target sequence, sensitivity differences between real-time PCR from whole blood and real-time PCR from serum were found to be negligible [8]. Chip-based molecular diagnostic approaches were tested as well but were found to be less sensitive compared to traditional real-time PCR performed in benchtop cyclers [7]. Acceptable sensitivity of *T. cruzi* detection based on real-time PCR has also been demonstrated for food containing crushed triatomes [5,6].

Although the first multicentric approaches with the aim of the standardization of the molecular diagnosis of Chagas disease were conducted more than 10 years ago [4], imperfect diagnostic accuracy of molecular diagnostic assays has remained a relevant issue [13,14,15,16], as recently described in more detail [17]. Further optimization is therefore an ongoing need that has to be addressed. To ensure the best available quality for infectious disease diagnostics, the European Union has released Regulation (EU) 2017/746. This regulation demands the application of certified tests in European Union-affiliated laboratories, unless it can be proven by the diagnosing laboratory that applied in-house diagnostic products are qualitatively better than available commercial products.

In order to contribute to the ongoing optimization of the molecular diagnosis of *T. cruzi*, this study comparatively assessed two commercial German real-time PCR assays targeting *T. cruzi* DNA in human serum samples with high pre-test probability. One of these assays, the NDO real-time PCR (TIB MOLBIOL, Berlin, Germany; ref. no. 53-0755-96, later referred to as the TibMolBiol assay) is a patent-protected scheme whose performance characteristics have recently been summarized [17,18]. The other assay, the RealStar Chagas PCR Kit 1.0 (altona DIAGNOSTICS, Hamburg, Germany; order no. 611013, later referred to as the RealStar assay), targets kinetoplast DNA and cannot differentiate between *T. cruzi* and *T. rangeli*, as declared by the manufacturer. Both assays had been internally validated by the manufacturers for in vitro diagnostic use in line with Regulation (EU) 2017/746 as declared by the “Conformité Européenne—in vitro diagnostics” (CE-IVD) label; however, details of the respective assessments have not been made publicly available. Residual serum samples from remote areas of endemicity for both *T. cruzi* and *T. rangeli* in tropical Colombia were used for the assay comparison to ensure a sufficiently high pre-test probability for the assessment. In case of discrepant results, sequences of the amplicons were assessed in order to come to a decision on specific or non-specific amplification.

## 2. Materials and Methods

### 2.1. Residual Sample Materials Included in the Assessments

A total of 518 serum samples collected from inhabitants of remote Colombian high-endemicity settings for Chagas disease, comprising the settlements Ahuyamal (Department Cesar), Ashintuwka, Cherua (both Department La Guajira), Dungakare, Sabannah de Higuieron, Seminke, Surimena and Tezhumake (all Department Cesar) and Sierra Nevada de Santa Marta, were included in the assessment, so a high pre-test probability for positive samples could be considered guaranteed, although the individual disease status of the individual donors was partly unknown. Neither serological nor molecular diagnostic results from previous assessments were continuously available for all samples included in the performed comparative test assessment without a reference standard. However, as assumed from previous local assessments [17,18], a regional prevalence of *Trypanosoma* spp. infections with circulating pathogen DNA in blood serum ranging from 10 to 30 percent was expected for the assessed study population. So, a high two-digit or low three-digit number of positive test results was considered likely.

The samples were collected a few months to several years prior to the assessment in the course of various studies, including a published one [17] and several yet-unpublished ones. Residual sample materials had been stored at −80 °C to ensure the stability of the target DNA. A reference standard for the comparative testing was not available. The female/male ratio of the individuals from whom the serum samples had been taken was nearly balanced at 1:1.2. The mean age ± standard deviation was 22.3 ± 18.3 years, and the median age (interquartile range) was 15 (8.34) years (Table 1).

In addition to the patients’ samples, nucleic acid extractions of the six discrete typing units (DTUs) of *T. cruzi*, as described recently [19], were included in the comparative real-time PCR assessment. The DTU DNA was a donation from the University del Rosario, Bogota, Colombia, and had been extracted from cultured epimastigote cells, as described by the donator elsewhere [4,15,20].

### 2.2. Nucleic Acid Extraction

Nucleic acid extraction was conducted applying the RTP Pathogen Kit (Stratec Molecular, Birkenfeld, Germany) with 200µL serum volumes, as described by the manufacturer. The eluates were stored at −80 °C prior to real-time PCR analysis.

### 2.3. Applied Real-Time PCR Assays

All eluates were assessed with the *T. cruzi*-specific NDO real-time PCR (TIB MOLBIOL, Berlin, Germany; ref. no. 53-0755-96, later referred to as the TibMolBiol assay) including the phocid herpes-virus-based PhHV extraction control (ref. 66-0901-96). The real-time PCR was run with the lyophilized 1-step RT-PCR Polymerase Mix (Cat-No 90-9999-96). The assay is a patent-protected scheme, as recently summarized in [17], targeting kinetoplast minicircle DNA (e.g., GenBank accession number U07846.1). In comparison to the recent publication [17], the probe sequence had been adapted to 5′-FAM-TCG+AACCCC+ACCTCC-BHQ-1-3′ with the “+” symbol marking locked nucleic acid (LNA) bases. As a competitor assay, the RealStar Chagas PCR Kit 1.0 (altona DIAGNOSTICS, Hamburg, Germany; order no. 611013, later referred to as the RealStar assay) was applied, which targets the kinetoplast DNA, does not differentiate between *T. cruzi* and *T. rangeli* and contains a manufacturer-provided internal reaction control. Both commercial hybridization probe (also called “TaqMan”) real-time PCR assays were run on Rotor-Gene Q cyclers (Qiagen, Hilden, Germany) controlled by the Rotor-Gene Q Series software version 2.3.5 (Qiagen, Hilden, Germany), exactly as described by the manufacturer. No deviations from the manufacturers’ demands regarding the interpretation of the real-time PCR signals were introduced. Cycle threshold (Ct) values of positive samples were recorded and compared for true-positive as well as cross-reacting specimens.

### 2.4. Interpretation of the Results

Concordantly positive and negative results were accepted as “true” positive and negative for *T. cruzi*. As described recently [17], Sanger sequencing results discriminating between *T. cruzi* and *T. rangeli* could be successfully obtained after inclusion of the amplicons in vector plasmids for a minor number of residual sample materials in order to decide on true or false *T. cruzi* detections. The real-time PCR amplicons of the remaining discordant samples were subjected to nanopore sequencing at the Center for Biotechnology (CeBiTec), Bielefeld University, Germany. Barcoded libraries were prepared using the SQK-LSK109 kit with EXP-NBD196, and the pool was run on an R9.4.1 flow cell. The obtained reads were matched with publicly available *T. cruzi* and *T. rangeli* sequences using megablast with standard parameters and a best-hit approach. Thereby, species identity was pragmatically attributed to the species with the higher proportion of matching reads. Due to the lack of cut-offs for the diagnosis of co-infection with *T. cruzi* and *T. rangeli*, this hypothetical situation was not considered for this simplified interpretation approach and so mono-infections due to either *T. cruzi* or *T. rangeli* were assumed in all instances.

### 2.5. Statistics

In line with the straightforward study aim, only descriptive statistics were conducted. Sensitivity and specificity for the identification of circulating *T. cruzi*-specific DNA were calculated based on the aforementioned assumptions.

### 2.6. Ethics

Ethical clearance was provided by the Medical Association of Hamburg, Germany, (reference number: WF-011/19, obtained on 11 March 2019), allowing anonymous use of residual sample materials for test comparison purposes without informed consent. The assessments were conducted according to the guidelines of the Declaration of Helsinki.

## 3. Results

### 3.1. Comparison of the Results of the Assays with the Nucleic Acid Extractions of the Six Discrete Typing Units (DTUs) of T. cruzi

**Figure A1:**
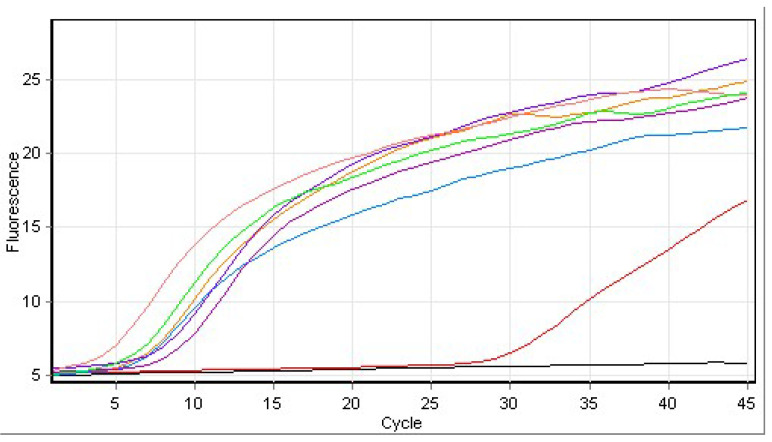
Exemplary visualization of recorded amplification curves. Here: amplification with samples containing DTU DNA at high concentrations according to the RealStar assay. Positive control amplification can be seen at cycle threshold 29.

When applied with nucleic acid extractions of the six DTUs of *T. cruzi* from cell culture, so high concentrations of parasite DNA were guaranteed, both the TibMolBiol assay and the RealStar assay detected 100% (6/6) of the DTUs. High DNA concentrations within the specimens were confirmed by very low cycle threshold (Ct) values, as indicated in Table 2. Amplification curves with the RealStar assay are exemplarily visualized in the Appendix A Figure A1.

### 3.2. Comparison of the Results of the Assays with the Nucleic Acid Extractions from the Patient Sera

Out of 518 assessed sera, 400 (77.2%) showed concordantly negative results and 89 (17.2%) showed concordantly positive results, resulting in a total of 489 (94.4%) concordant results. Out of the 29 (5.6%) discordant results, 6 were positive in the TibMolBiol assay only, and 23 were positive in the RealStar assay only. For three out of the six samples with additional positive real-time PCR signals in the TibMolBiol assay, Sanger sequencing [17] confirmed the species diagnosis of *T. cruzi*, and so they were considered true-positive in the TibMolBiol assay and false-negative in the RealStar assay. For 6 out of 23 samples that were additionally positive in the RealStar assay, Sanger sequencing [17] confirmed the species diagnosis of *T. rangeli*, and so the results do not indicate *T. cruzi*, confirming the negative *T. cruzi*-specific results in the TibMolBiol assay as true negative. The real-time PCR amplicons of the remaining 20 discordant samples were subjected to nanopore sequencing as stated above. When comparing the reads with publicly available *T. cruzi* and *T. rangeli* sequences, including consecutive attribution of species identity to the species with more matching reads, the obtained ratios of the better-matching species and the weaker-matching species ranged from a minimum of 1.012:1 (50.3% vs. 49.7%) to a maximum of 24:1 (96.0% vs. 4.0%). Accepting the associated residual uncertainty, 2 out of the 20 samples (10%) were attributed to *T. cruzi* and 18 out of 20 samples (90%) to *T. rangeli*, increasing the total number of *T. cruzi* detections to 94 and the total number of *T. rangeli* detections to 24. Thereby, the two additional *T. cruzi* DNA-containing samples, as identified based on the nanopore sequencing results, had been missed by the TibMolBiol assay but were correctly identified by the RealStar assay. Among the samples containing *T. rangeli*-specific DNA, three cross-reactions with the TibMolbiol assay but not with the RealStar assay were observed in spite of the co-specificity for *T. cruzi* and *T. rangeli* of the latter, as claimed by its manufacturer. Based on the abovementioned results and assumptions, the resulting sensitivity and specificity for the specific detection of *T. cruzi* were 97.9% (92/94) and 99.3% (421/424) with the TibMolBiol assay and 96.8% (91/94) and 95.0% (403/424) with the RealStar assay, respectively (Table 3).

### 3.3. Comparison of the Cycle Threshold (Ct) Values between the Assays

When focusing on the measured cycle threshold (Ct) values, rather low Ct values were recorded for the RealStar assay compared to the TibMolBiol assay. In contrast, there was no obvious difference in the Ct value distribution between samples containing *T. cruzi* DNA and samples containing *T. rangeli* DNA for both assays. Details are provided in Table 4.

## 4. Discussion

This study was performed for the comparison of two commercially available real-time PCR assays for the diagnosis of the DNA of *T. cruzi*, the causative agent of Chagas disease, in human serum. Residual samples from individuals living in a high-endemicity setting were applied in a head-to-head comparison, so a high pre-test probability was expected [17]. Both assays showed a comparable diagnostic accuracy for the detection of *T. cruzi* in human serum with robust sensitivity and specificity for the TibMolBiol assay as well as a similarly good sensitivity with only a slightly worse specificity for the RealStar assay. In addition to minor accuracy differences as well as stochastically distributed real-time PCR results in the case of pathogen DNA density close to the diagnostic detection threshold, the fact that the RealStar assay also targets DNA of the phylogenetically closely related but apathogenic species *T. rangeli* accounted for most of the observed discrepancies between the assays.

Focusing on the cycle threshold values of correctly identified *T. cruzi*-containing samples as well as cross-reactions with *T. rangeli*, the identification of non-specific reactions by higher Ct values was unfeasible with both assays, because no obvious differences between *T. cruzi*-specific and non-specific reactions were seen. This finding confirms the general challenge of the high phylogenetic similarity of the species, making their discrimination via real-time PCR difficult.

Both real-time PCR assays correctly identified DNA of the six discrete typing units (DTUs) of *T. cruzi* described in the literature [19] as well, suggesting high conservation of the sequence targets and thus applicability in different geographic regions. The DTU assessment was performed with highly concentrated pathogen DNA, as reflected by very low measured Ct values, although absolute quantification data from the extracted culture materials were not available. To avoid DNA contamination in the laboratory, such high concentrations are generally undesirable. In this case, the assessment without further dilution steps was performed to exclude a lack of oligonucleotide binding even in spite of excess amounts of target DNA. Further focus on the topic of DTUs, which is still an important matter of debate in both epidemiological and basic research [4,15,20,21,22], would have been beyond the scope of this test comparison, which was primarily meant to simulate routine-like diagnostic conditions.

This study was performed as a comparative head-to-head assessment of two CE-IVD-labeled commercial real-time PCR assays for in vitro diagnostics without a reference standard. Conventional serology based on two different assays, which is suggested as a reference standard for Chagas disease diagnostics by the World Health Organization (WHO) and the Pan American Health Organization (PAHO), was not used as a reference standard for this assessment, because (a) a positive serological result indicates previous contact with the pathogen but not necessarily circulating *T. cruzi* DNA in blood serum, as tested in this assessment, and (b) negative serology does not necessarily exclude abundance of circulating *T. cruzi* DNA in serum, either due to very early infections or due to a lacking immune response in the case of immunological disorders. Adding another molecular assay and considering this particular assay as a consensus approach would not have resolved the residual uncertainty either. To serve as a true “gold standard” for the study, this consensus approach would require a sensitivity and specificity of 100% for the detection of *T. cruzi* DNA in human serum, a prerequisite that is hardly fulfilled by any diagnostic assay available.

This study has a number of limitations. First, a lack of funding for sequence-based confirmation of parasite species identity for all and not just for the discrepant real-time PCR results made it necessary to assume the abundance of *T. cruzi*-specific DNA in the case of concordantly positive real-time PCR results. However, the study results indicate minor cross-reactivity with *T. rangeli* for the TibMolBiol real-time PCR, although high specificity of this assay, as previously shown [17,18], was still generally confirmed. Accordingly, it has to be assumed that the true specificity of both real-time PCR assays is even slightly lower than that postulated due to some *T. rangeli* infections within the non-controlled group of 89 concordantly positive samples. Second, and as a more general issue [23], even negative real-time PCR results in both real-time PCRs can never definitely exclude the abundance of pathogen DNA below the diagnostic detection threshold. So, sensitivity—as reported in this study—only reflects parasitemia resulting in target DNA concentrations above the limit of detection of the assessed real-time PCR assays, which may be the reason for the strikingly better sensitivity observed here than that reported in previous studies, in which real-time PCR results were compared to serology-based reference standards [12]. Third, the TibMolBiol assay was optimized for the applied nucleic acid extraction scheme [24] while the RealStar assay was not, potentially resulting in a minor advantage for the TibMolBiol assay in this assessment. Available sample volumes were insufficient to allow extraction with different nucleic acid extraction assays. However, real-time PCR from identical nucleic acid extraction eluates in close temporal association at least ensured comparable reaction conditions for both assays. Additionally, a recent assessment indicated that the actual impact of the chosen nucleic acid extraction assay may be low as long as modern commercial extraction schemes for serum samples are applied [24]. Fourth, the assays included in the assessment did not comprise a comprehensive spectrum of globally available diagnostic real-time PCR assays targeting *T. cruzi* [10,12], just products regionally available in Germany, where the comparison was conducted. Accordingly, the abovementioned Dutch *T. cruzi*-specific real-time PCR assay RealCycler CHAG kit (EMELCA Bioscience), whose diagnostic accuracy estimates are shown above, was not part of the assessment, because the inclusion of international diagnostic products would have been beyond the funding limits of this assessment. Finally, the partly poor quality of available *T. cruzi* and *T. rangeli* sequences from the databases made the attribution of the nanopore sequence reads challenging, and the lack of cut-offs made any reliable identification or exclusion of co-infections with *T. cruzi* and *T. rangeli* unfeasible. The resulting residual uncertainty, which could not be further technically reduced, suggests the need for a careful interpretation of minor observed differences between the two compared assays, although the postulation of similar diagnostic accuracy with moderately better specificity of the TibMolBiol assay still seems justified. The same problem, however, also applies to Sanger sequencing results, because amplicon sequencing based on next-generation sequencing is known to be even more sensitive than Sanger sequencing for the detection of variants occurring in minor proportions, as known from genotypic HIV resistance testing [25]. The decision to switch from Sanger sequencing to nanopore sequencing was purely driven by the greater cost-efficient applicability of the latter and not by reliability concerns.

## 5. Conclusions

Both assessed real-time PCR assays showed similar diagnostic accuracy for the identification of *T. cruzi*-specific DNA in human blood serum, with a slightly better specificity of the TibMolBiol assay. In areas where co-circulation of *T. cruzi* and *T. rangeli* infections occurs, the more pronounced co-amplification of *T. rangeli*-specific DNA by the RealStar assay presents a diagnostic disadvantage compared to the TibMolBiol assay. However, the similar sensitivity of the RealStar assay and the TibMolBiol assay and the restriction of false-positive signals to *T. rangeli* DNA-containing samples as observed in this study make both assays useful for settings in which co-occurrence of *T. rangeli* infections only plays a minor role or is not observed at all.

## Figures and Tables

**Table 1:** Summary of the assessed Indigenous study population, comprising 518 individuals from a region with a high prevalence of *Typanosoma* spp. DNA in peripheral blood.

Female/male ratio	1:1.2
Age in years (mean ± standard deviation; median (interquartile range))	22.3 ± 18.3; 15 (8.34)
Study locations within the Sierra Nevada de Santa Marta, Colombia	Ahuyamal (Department Cesar), Ashintuwka, Cherua (both Department La Guajira), Dungakare, Sabannah de Higuieron, Seminke, Surimena and Tezhumake (all Department Cesar)

**Table 2:** Cycle threshold (Ct) values as recorded with nucleic acid extractions of the six DTUs of *T. cruzi*.

	TibMolBiol Real-Time PCR		RealStar Real-Time PCR	
	Qualitative Result	Quantitative Result (Ct Value)	Qualitative Result	Quantitative Result (Ct Value)
*T. cruzi* DTU I	Positive	7.38	Positive	5.55
*T. cruzi* DTU II	Positive	6.27	Positive	5.77
*T. cruzi* DTU III	Positive	6.89	Positive	6.37
*T. cruzi* DTU IV	Positive	3.85	Positive	2.80
*T. cruzi* DTU V	Positive	7.56	Positive	6.97
*T. cruzi* DTU VI	Positive	5.42	Positive	5.20

Ct = cycle threshold.

**Table 3:** Flowchart of the diagnostic results and their interpretation.

Comparative assessment of 518 consecutive serum samples with both real-time PCR assays (TibMolBiol and RealStar)			
↓			
89 concordantly positive results	6 samples positive in the TibMolBiol assay only	23 samples positive in the RealStar assay only	400 concordantly negative results
↓	↓	↓	↓
Accepted as presumedly positive for the abundance of *T. cruzi* DNA without further assessments	Sequence-based identification of 3 samples positive for *T. cruzi* DNA and 3 samples positive for *T. rangeli* DNA	Sequence-based identification of 2 samples positive for *T. cruzi* DNA and 21 samples positive for *T. rangeli* DNA	Accepted as presumedly negative for the abundance of *T. cruzi* DNA without further assessments
Resulting sensitivity calculations for the identification of the abundance of *T. cruzi*-specific DNA	97.9% (92/94) with the TibMolBiol assay and 96.8% (91/94) with the RealStar assay		
Resulting specificity calculations for the identification of the abundance of *T. cruzi*-specific DNA	99.3% (421/424) with the TibMolBiol assay and 95.0% (403/424) with the RealStar assay		

**Table 4:** Cycle threshold (Ct) values (mean, standard deviation (SD)) for correctly identified samples and for cross-reactions with *T. rangeli*-positive samples in the TibMolBiol assay and in the RealStar assay.

	Correctly Identified *T. cruzi*-Positive Samples in the TibMolBiol Assay (n = 92)	Correctly Identified *T. cruzi*-Positive Samples in the RealStar Assay (n = 91)	Cross-Reactions with *T. rangeli*-Positive Samples in the TibMolBiol Assay (n = 3)	Cross-Reactions with *T. rangeli*-Positive Samples in the RealStar Assay (n = 21)
Mean value of the Ct values	34.2	25.4	35.0	25.1
Standard deviation (SD) of the Ct values	3.6	2.5	1.7	3.0

Ct = cycle threshold.

## Data Availability

All relevant data are provided in the manuscript. Raw data can be provided at reasonable request.

## References

[1] Andrade D.V., Gollob K.J., Dutra W.O. (2014). Acute chagas disease: New global challenges for an old neglected disease. PLoS Negl. Trop. Dis..

[2] Moreira O.C., Ramírez J.D., Velázquez E., Melo M.F., Lima-Ferreira C., Guhl F., Sosa-Estani S., Marin-Neto J.A., Morillo C.A., Britto C. (2013). Towards the establishment of a consensus real-time qPCR to monitor *Trypanosoma cruzi* parasitemia in patients with chronic Chagas disease cardiomyopathy: A substudy from the BENEFIT trial. Acta Trop..

[3] Schijman A.G., Alonso-Padilla J., Longhi S.A., Picado A. (2022). Parasitological, serological and molecular diagnosis of acute and chronic Chagas disease: From field to laboratory. Mem. Inst. Oswaldo Cruz.

[4] Schijman A.G., Bisio M., Orellana L., Sued M., Duffy T., Mejia Jaramillo A.M., Cura C., Auter F., Veron V., Qvarnstrom Y. (2011). International study to evaluate PCR methods for detection of *Trypanosoma cruzi* DNA in blood samples from Chagas disease patients. PLoS Negl. Trop. Dis..

[5] Mattos E.C., Meira-Strejevitch C.D.S., Marciano M.A.M., Faccini C.C., Lourenço A.M., Pereira-Chioccola V.L. (2017). Molecular detection of *Trypanosoma cruzi* in acai pulp and sugarcane juice. Acta Trop..

[6] Finamore-Araujo P., Faier-Pereira A., Ramon do Nascimento Brito C., Gomes Peres E., Kazumy de Lima Yamaguchi K., Trotta Barroso Ferreira R., Moreira O.C. (2021). Validation of a novel multiplex real-time PCR assay for Trypanosoma cruzi detection and quantification in açai pulp. PLoS ONE.

[7] Rampazzo R.C.P., Graziani A.C., Leite K.K., Surdi J.A., Biondo C.A., Costa M.L.N., Jacomasso T., Cereda M., De Fazio M., Bianchessi M.A. (2019). Proof of Concept for a Portable Platform for Molecular Diagnosis of Tropical Diseases: On-Chip Ready-to-Use Real-Time Quantitative PCR for Detection of *Trypanosoma cruzi* or *Plasmodium* spp. J. Mol. Diagn..

[8] Melo M.F., Moreira O.C., Tenório P., Lorena V., Lorena-Rezende I., Júnior W.O., Gomes Y., Britto C. (2015). Usefulness of real time PCR to quantify parasite load in serum samples from chronic Chagas disease patients. Parasit. Vectors.

[9] Ferreira Filho J.C.R., Braz L.M.A., Andrino M.L.A., Yamamoto L., Kanunfre K.A., Okay T.S. (2019). Mitochondrial and satellite real time-PCR for detecting *T. cruzi* DTU II strain in blood and organs of experimentally infected mice presenting different levels of parasite load. Exp. Parasitol..

[10] Abras A., Ballart C., Llovet T., Roig C., Gutiérrez C., Tebar S., Berenguer P., Pinazo M.J., Posada E., Gascón J. (2018). Introducing automation to the molecular diagnosis of *Trypanosoma cruzi* infection: A comparative study of sample treatments, DNA extraction methods and real-time PCR assays. PLoS ONE.

[11] Duffy T., Bisio M., Altcheh J., Burgos J.M., Diez M., Levin M.J., Favaloro R.R., Freilij H., Schijman A.G. (2009). Accurate real-time PCR strategy for monitoring bloodstream parasitic loads in chagas disease patients. PLoS Negl. Trop. Dis..

[12] Longoni S.S., Pomari E., Antonelli A., Formenti F., Silva R., Tais S., Scarso S., Rossolini G.M., Angheben A., Perandin F. (2020). Performance Evaluation of a Commercial Real-Time PCR Assay and of an In-House Real-Time PCR for *Trypanosoma cruzi* DNA Detection in a Tropical Medicine Reference Center, Northern Italy. Microorganisms.

[13] Qvarnstrom Y., Schijman A.G., Veron V., Aznar C., Steurer F., da Silva A.J. (2012). Sensitive and specific detection of *Trypanosoma cruzi* DNA in clinical specimens using a multi-target real-time PCR approach. PLoS Negl. Trop. Dis..

[14] Piron M., Fisa R., Casamitjana N., López-Chejade P., Puig L., Vergés M., Gascón J., Gómez i Prat J., Portús M., Sauleda S. (2007). Development of a real-time PCR assay for *Trypanosoma cruzi* detection in blood samples. Acta. Trop..

[15] Ramírez J.C., Cura C.I., da Cruz Moreira O., Lages-Silva E., Juiz N., Velázquez E., Ramírez J.D., Alberti A., Pavia P., Flores-Chávez M.D. (2015). Analytical Validation of Quantitative Real-Time PCR Methods for Quantification of *Trypanosoma cruzi* DNA in Blood Samples from Chagas Disease Patients. J. Mol. Diagn..

[16] De Winne K., Büscher P., Luquetti A.O., Tavares S.B., Oliveira R.A., Solari A., Zulantay I., Apt W., Diosque P., Monje Rumi M. (2014). The *Trypanosoma cruzi* satellite DNA OligoC-TesT and Trypanosoma cruzi kinetoplast DNA OligoC-TesT for diagnosis of Chagas disease: A multi-cohort comparative evaluation study. PLoS Negl. Trop. Dis..

[17] Kann S., Kunz M., Hansen J., Sievertsen J., Crespo J.J., Loperena A., Arriens S., Dandekar T. (2020). Chagas Disease: Detection of Trypanosoma cruzi by a New, High-Specific Real Time PCR. J. Clin. Med..

[18] Kann S., Zabala-Monterroza W., García C., Concha G., Landt O., Hahn A., Weinreich F., Frickmann H. (2022). Comparison of the Influence of Different Nucleic Acid Extraction Assays on the Sensitivity of *Trypanosoma cruzi*-Specific Real-Time PCR. Microorganisms.

[19] Probst M.C., de Fátima Alheiros Dias Melos M., Parada Pavoni D., de Ornelas Toledo M.J., Silva Galdino T., Alves Brandão A., Britto C., Krieger M.A. (2021). A new *Trypanosoma cruzi* genotyping method enables high resolution evolutionary analyses. Mem. Inst. Oswaldo Cruz.

[20] Hernández C., Cucunubá Z., Flórez C., Olivera M., Valencia C., Zambrano P., León C., Ramírez J.D. (2016). Molecular Diagnosis of Chagas Disease in Colombia: Parasitic Loads and Discrete Typing Units in Patients from Acute and Chronic Phases. PLoS Negl. Trop. Dis..

[21] Rodrigues-Dos-Santos Í., Melo M.F., de Castro L., Hasslocher-Moreno A.M., do Brasil P.E.A.A., Silvestre de Sousa A., Britto C., Moreira O.C. (2018). Exploring the parasite load and molecular diversity of *Trypanosoma cruzi* in patients with chronic Chagas disease from different regions of Brazil. PLoS Negl. Trop. Dis..

[22] Zingales B. (2018). *Trypanosoma cruzi* genetic diversity: Something new for something known about Chagas disease manifestations, serodiagnosis and drug sensitivity. Acta Trop..

[23] Hahn A., Podbielski A., Meyer T., Zautner A.E., Loderstädt U., Schwarz N.G., Krüger A., Cadar D., Frickmann H. (2020). On detection thresholds-a review on diagnostic approaches in the infectious disease laboratory and the interpretation of their results. Acta Trop..

[24] Kann S., Dib J.C., Aristizabal A., Mendoza G.C., Lacouture H.D.S., Hartmann M., Frickmann H., Kreienbrock L. (2022). Diagnosis and Prevalence of Chagas Disease in an Indigenous Population of Colombia. Microorganisms.

[25] Tzou P.L., Ariyaratne P., Varghese V., Lee C., Rakhmanaliev E., Villy C., Yee M., Tan K., Michel G., Pinsky B.A. (2018). Comparison of an *In Vitro* Diagnostic Next-Generation Sequencing Assay with Sanger Sequencing for HIV-1 Genotypic Resistance Testing. J. Clin. Microbiol..

